# On the Cure of the Croup, Obstinate Coughs, Hooping Cough, &c. by Liver of Sulphur (Sulphat of Potass or Soda)

**Published:** 1816-11

**Authors:** 


*For the London Medical and Physical Journal.*

### *On the Cure of the Croup, obstinate Coughs, Hooping Cough, &c. by Liver of Sulphur (Sulphat of Potass or Soda).*— From our Parisian Correspondent.

THE author of this discovery, whose name is not yet given to the public, asserts, that he had never known it fail in the croup and hooping cough. Experience has proved that, though not infallible, it is a highly important recipe, in the latter disorder especially, as well as in old and confirmed phthisical coughs. The author proposes the liver of sulphur, newly prepared, to be administered in doses of from six to ten grains, night and morning, diminishing the dose according to the progress of recovery; and when this becomes very sensible, it is only to be taken in the morning. It is not the age of the patient which induces the author to augment the dose, but simply the violence of the disorder.

In administering the dose to children, the author recommends the liver of sulphur being mixed with honey, in which the finger is to be dipped, and put in the infant's mouth, until it has taken it completely. If the patient rejects it, a new dose must instantly be administered. It may also be given in a table-spoonful of milk, in diluted simple syrup or as a bolus. If the medical attendant have any doubt of the administration of the medicine, he should see it taken himself.

In the croup, says the author, the patient feels sensibly better, even the second day; but the medicine must be regularly continued, even a few days after the disorder has wholly disappeared.

Infants at the breast may continue to suck while taking the medicine, but others are directed to take only light food and nourishing liquids.

The author recommends the remedy, not only as a cure, but as a preservative from the croup, and he employs it on the slightest symptom of the disorder; but, when it cannot instantly be procured, he recommends topical bleeding, which allays the violence of the symptoms until the liver of sulphur can be administered.

Dr. Wesener, of Dulmen, cites the following cases of its efficacy. Dr. W.'s son, aged three years, of a lively irritable temper, had laboured under a violent cough three weeks: the voice was hoarse, and the cough dry, which continued ordinarily all night. M. Wesener dissolved thirty-six grains of liver of sulphur in two drachms of distilled water with one one ounce of syrup of marsh mallows. He administered about a drachm (containing from three to four grains of liver of sulphur), at nine in the morning—the stomach rejected half. At bed-time, Dr. W. administered two drachms, which remained on the stomach. The child was considerably better during the night, for it had only two fits of coughing, which had even ceased to be dry. The medicine was continued two days, with sensible benefit; but, the child taking an aversion to the liver of sulphur, it was suspended the third day, and in the night the cough returned. The medicine was, therefore, resumed during two days, in doses of two drachms morning and evening. On the third day only one dose was administered at bed-time, and the cough entirely ceased.

The daughter of a labouring man was afflicted with the hooping-cough, a fortnight. The fits came on regularly every half hour, and they were so violent that the blood issued from the nose and mouth. One drachm of the remedy was administered four times a day. In five or six days, the little patient, though constantly exposed to the north wind, had only five fits each day. The medicine was continued four times a day, in doses of two drachms; and on the twelfth day the parents found the child so perfectly recovered that they desisted from administering the remedy altogether.

A weaver, aged twenty-five, had an obstinate phthisical cough, which was most troublesome during the night, and was so dry and painful that each fit was followed by vomiting and mucous excretion. The patient took every two hours six grains of liver of sulphur, in a table-spoonful of the infusion of angelica. The cough, though equally frequent, became less violent, and attended with the copious expectoration of mucus. The third day, the medicine was continued, with the addition of half a grain of extract of henbane (hyoscyamus) dissolved in vin. antim. The fifth day the patient was cured.

These cases prove the powerful action of liver of sulphur on the mucous membranes of the tracheal artery, and on the lungs. It should, however, be recollected that it must be administered with great care. Dr. Orfila, in his learned treatise on Poisons, has proved that sulphat of potass is a corrosive poison, which, introduced into the stomach of a middle-sized dog, in the dose of one drachm, killed him in a few hours, when vomiting was prevented. It is, consequently, particularly to be observed not to increase the doses prescribed.

It

It may be necessary to remark, that the first action induced by the liver of sulphur is to whiten the lips and the mouth, to produce a degree of heat in the stomach, and sometimes vomiting of viscous matter, tinged green by the remedy.

---

*For the London Medical and Physical Journal.*

### Mr. Woodham *in Answer to Mr. Walker.*

