# Corrigenda

**Published:** 1816-12

**Authors:** 


					u. 373, throughout the article, for
sulpbat read sulphurct;

				

